# Characterization and Aerosolization Performance of HydroxyPropyl-Beta-Cyclodextrin Particles Produced Using Supercritical Assisted Atomization

**DOI:** 10.3390/polym13142260

**Published:** 2021-07-09

**Authors:** Hsien-Tsung Wu, Yao-Hsiang Chuang, Han-Cyuan Lin, Liang-Jung Chien

**Affiliations:** Department of Chemical Engineering, Ming Chi University of Technology, 84 Gungjuan Rd., Taishan Dist., New Taipei City 24301, Taiwan; M07138123@mail2.mcut.edu.tw (Y.-H.C.); M08138104@mail2.mcut.edu.tw (H.-C.L.); ljchien@mail.mcut.edu.tw (L.-J.C.)

**Keywords:** supercritical assisted atomization, hydroxypropyl-β-cyclodextrin, leucine, in vitro aerosolization

## Abstract

In this study, hydroxypropyl-beta-cyclodextrin (HP-β-CD) particles were produced using supercritical assisted atomization (SAA) with carbon dioxide as the spraying medium or co-solute and aqueous ethanol solution as the solvent. The effects of several key factors on the morphology and size of the HP-β-CD particles were investigated. These factors included the solvent effect, temperatures of the precipitator and saturator, concentration of the HP-β-CD solution, and flow rate ratio of carbon dioxide to the HP-β-CD solution. The conducive conditions for producing fine spherical particles were 54.2% (*w/w*) aqueous ethanol as the solvent; precipitator and saturator temperatures of 373.2 K and 353.2 K, respectively; a flow rate ratio of carbon dioxide to HP-β-CD solution of 1.8; and low concentrations of HP-β-CD solution. The addition of leucine (LEU) enhanced the aerosol performance of the HP-β-CD particles, and the fine particle fraction (*FPF*) of the HP-β-CD particles with the addition of 13.0 mass% LEU was 1.8 times higher than that of the HP-β-CD particles without LEU. This study shows that LEU can act as a dispersion enhancer and that HP-β-CD particles produced using SAA can be used as pulmonary drug carriers.

## 1. Introduction

Cyclodextrins (CDs) are cyclic oligosaccharides consisting of covalently linked glucopyranose rings. Naturally occurring CDs include α-, β-, and γ-CDs, which comprise six, seven, and eight glucopyranose units, respectively. CDs adopt the shape of a truncated cone, due to the chair structure of the glucopyranose units, where the hydroxyl groups are oriented to the cone exterior with the primary hydroxyl groups (hydrophobic character) of the sugar residues at the narrow edge of the cone, and the secondary hydroxyl groups at the wider edge [1]. The hydrophobic cavity of CD acts as a molecular container to entrap guest molecules to form inclusion complexes. Many advantages of drug complexes containing cyclodextrin have been reported in the literature, including increased solubility, enhanced bioavailability, improved stability, and different novel drug delivery routes [2].

The water-soluble CD derivatives of pharmaceutical interest include the hydroxypropyl derivatives of β-CD and γ-CD (HP-β-CD and HP-γ-CD), randomly methylated β-CD (RM-β-CD), and sulfobutylether β-CD (SBE-β-CD). HP-β-CD and SBE-β-CD are cited in the FDA’s list of inactive pharmaceutical ingredients. HP-β-CD is present in a range of commercially available injectable formulations [3] and has been demonstrated to be safe for application in human airway epithelial Calu-3 cells in vitro, demonstrating its potential for use in commercial dry powders for inhalation formulations [4,5,6]. In addition, HP-β-CD can form water-soluble complexes with poorly water-soluble drugs and can increase the drug molecule permeability and bioavailability [7,8,9].

Conventional methods for obtaining CD microparticles include grinding [10], spray drying, freeze drying [11], and co-evaporation [12]. However, these methods do not assure the efficient control of the particle size and can cause thermal or chemical degradation and low reproducibility among different batches. Supercritical carbon dioxide (scCO_2_) is a benign working medium that plays versatile roles in particle formation technologies, including the rapid expansion of supercritical solutions (RESS; scCO_2_ as a function of the solvent), supercritical antisolvent (SAS; scCO_2_ as a function of the antisolvent), and supercritical assisted atomization (SAA; scCO_2_ as a function of the spraying medium or the co-solute). RESS and SAS were not adopted in this study because of the solubility limitation of the applied solute in scCO_2_ and the aqueous solvent in antisolvent of scCO_2_, respectively. SAA has been applied to aqueous and organic solvent systems to achieve a stable, high-yielding, and easily scalable process [13,14,15]. Reverchon and Antonacci [16] first reported α-CD and HP-β-CD micronization by SAA using water as the solvent. The present study further investigated the effects of aqueous ethanol as the solvent and SAA operation parameters on the morphology and size of HP-β-CD particles. The production of CD carrier particles for dry powder inhaler (DPI) formulations by one-step SAA is a novel method. Therefore, in this study, HP-β-CD carrier particles were produced by the SAA process using varying amounts of aqueous ethanol as the solvent and adding leucine (LEU) to enhance the aerosolization of the composite particles. The aim of this study was to determine the optimal parameters for the SAA process and the optimal addition amount of LEU for preparing HP-β-CD carrier particles with excellent aerosol performance.

## 2. Materials and Methods

### 2.1. Materials

HP-β-CD (99.9% purity) and _L_-leucine (99.9% purity) were purchased from Sigma Aldrich, Eschenstrasse, Taufkirchen, Germany. Ethanol (99.9% purity, high-performance liquid chromatography grade) was purchased from Acros, USA. Carbon dioxide (99.9% purity) and nitrogen (99.9% purity) were purchased from Yung-Ping Gas Co., Taipei, Taiwan. All chemicals were used without further purification. A Millipore Milli-Q water purification system was used to obtain deionized water with a resistivity of 18 MΩ-cm at 25 °C.

### 2.2. Production of HP-β-CD Carrier Particles

A schematic diagram of the SAA apparatus and experimental procedure has been provided elsewhere [17]. The apparatus is consisted by a saturator (Model: 7973, 24 cm^3^, Applied Separations, Allentown, PA, USA), a precipitator, a separator (CT10/−50 °C, Firstek, Taipei, Taiwan), and three feeding lines, including the HP-β-CD solution, CO_2_, and N_2_. Two high-pressure liquid pumps were used to deliver CO_2_ (NP-KX-540, Nihon Seimitsu Kagaku Co., Hon-cho, Kawaguchi, Japan) and the HP-β-CD solution (PU-1580, JASCO, Hachioji, Tokyo, Japan). Using a mass controller (251-FKASBYAA, Porter, Hatfield, PA, USA), the N_2_ flow was controlled from a cylinder, heated in an electric heat exchanger (series 93, Watlow, St. Louis, MO, USA), and, thereafter, sent to the precipitator to assist in the evaporation of the liquid droplets. 

The experimental procedure is briefly described as follows. The preset ethanol contents of the aqueous ethanol solution (EtOH%, *w/w*) as the solvent and concentrations of HP-β-CD (*C_HP_*) and leucine (*C_LEU_*) in the HP-β-CD solution were prepared and are presented in Table 1 and Table 2. The mixture was vigorously mixed with ultrasonication for 2 h to ensure a homogeneous solution for the feeding line of the HP-β-CD solution in the SAA process. The temperatures of the saturator (*T_S_*) and precipitator (*T_P_*), and volumetric flow rate of CO_2_, were also preset. The N_2_ flow rate was 1.0 Nm^3^/h. After achieving a steady state, the HP-β-CD solution was introduced into the saturator via a pre-heated water bath at a flow rate of 3 mL/min. The CO_2_ mixture that was dissolved in the HP-β-CD solution and obtained by the saturator was sprayed through an injection nozzle (inner diameter of 130 μm) to atomize the liquid droplets in the precipitator. After contact of the droplet solution with the heated N_2_ and the consequent evaporation of the solvent from the droplets, HP-β-CD particles were formed because of the supersaturation of the solute. The samples were collected from the precipitator and observed by field-emission scanning electron microscopy (FESEM, model 6500, JEOL, Akishima, Tokyo, Japan). 

The particle size distribution (*PSD*) of the HP-β-CD carrier particles was determined using a dynamic light scattering (DLS) particle analyzer (Zatasizer Nano ZS90, Malvern, UK). As described in the literature [16], the particles were suspended in petroleum oil at 293.15 K and ultrasonicated for 1 min. The *PSD* was calculated by applying the Mie theory, using a refractive index of HP-β-CD of 1.520 [18]. The arithmetic and mass-weighted mean particle diameters, *d_no_* and *d*_4,3_, respectively, were calculated from the equations $d_{no}=\sum_{i=1}^{i}x_{i}D_{i}$ and $d_{4,3}=\frac{\sum_{i=1}^{i}x_{i}D_{i}^{4}}{\sum_{i=1}^{i}x_{i}D_{i}^{3}}$, where *x* represents the number fraction of the particles. All precipitation experiments were performed in triplicate (*n* = 3). 

### 2.3. Solid-State Characterization

The X-ray diffraction (XRD) patterns of the product powders were recorded using an X’Pert Pro X-ray powder diffractometer (PANalytical, Almelo, Netherlands) between 2θ values of 5° and 50°, at a scan rate of 0.02°/s. The infrared (IR) spectra of the samples were recorded using a Fourier transform infrared (FTIR) spectrophotometer (Nicolet iS5, Thermo Scientific Inc., Waltham, MA, USA) with an attenuated total reflection (ATR) element. The IR spectra were recorded from 400 to 4000 cm^−1^. The leucine content in the HP-β-CD carrier particles produced through SAA was determined using a thermogravimetric analyzer (TGA, SDT Q600, TA, New castle, DE, USA) by gradually heating the sample in nitrogen (50 mL/min) from ambient temperature to 673 K at a rate of 10 K/min. 

The bulk density (ρ_b_) and tapped density (ρ_tap_) of the HP-β-CD powder were measured as descriptors of bulk powder cohesiveness and flow properties. Each HP-β-CD powder sample was filled in a 5 mL cylinder, and after recording the initial volume (bulk volume), the cylinder was tapped 1250 times (automated tap density analyzer, Auto top 02106-60-1, Quantachrome, Boyntons Beach, FL, USA) and the new volume was recorded (tapped volume). The number of taps was 1250, following the recommendation of the European Pharmacopoeia. The values of ρ_b_ and ρ_tap_ were calculated as the powder weight over the powder bulk volume (*n* = 0) and tapped volume (*n* = 1250), respectively. The flowability of the HP-β-CD powder was estimated using the Hausner ratio (*H_R_* = ρ_tap_/ρ_b_).

### 2.4. In Vitro Aerosol Performance Determined Using an Andersen Cascade Impactor

The aerosol behavior of the HP-β-CD carrier was determined using a HandiHaler (Boehringer Ingelheim, Ingelheim, Germany) coupled through an induction port (USP sampling inlet) to an Andersen cascade impactor (ACI, TE-20801, Tisch, Cleves, OH, USA), which was operated at a flow rate of 60 L/min. Hydroxypropyl methylcellulose capsules (size 3) were filled with the sample powder (20.0 ± 0.5 mg) and placed into the HandiHaler. The air flow rate for the ACI was adjusted to 60 L/min using a critical flow controller (TPK 2000, Copley, UK). This critical sonic flow was maintained (*P*_3_/*P*_2_ < 0.5) and the flow rate was assumed to be stable. The aerodynamic cut-off diameter of each stage of the ACI was calibrated by the manufacturer as follows: stage 1, 8.6 μm; stage 2, 6.5 μm; stage 3, 4.4 μm; stage 4, 3.3 μm; stage 5, 2.0 μm; stage 6, 1.1 μm; stage 7, 0.54 μm; and stage 8, 0.25 μm. The HP-β-CD carrier deposited at each stage was assayed using a gravity balance (ES225SM-DR, ±0.01 mg, Precisa, Switzerland). The emitted dose (*ED*) was determined as the difference between the initial mass of the powder loaded into the capsules (i.e., total dose, *TD*) and the remaining mass of the powder in the capsules following aerosolization. The *ED* fraction (*ED*, %) was used to express the percentage of *ED* based on the *TD* used. The fine particle dose (*FPD*) was defined as the quantity of particles with aerodynamic diameters <5 μm, and the dose deposited in stages 3‒8 of the ACI assay was obtained. The fine particle fraction (*FPF*, %) was expressed as the percentage of *FPD* to *TD*. In addition, to calculate the mass median aerodynamic diameter (*MMAD*), the cumulative percentage of the powder mass smaller than the stated aerodynamic diameter of the impactor stages from 1–8 was calculated and plotted against the effective cut-off diameter on a log probability plot. The *MMAD* of the aerosol particles was calculated using the method described by O’Shaughnessy and Raabe [19]. The in vitro aerosolization was evaluated in triplicate (*n* = 3) under ambient conditions, with a relative humidity of 40 ± 5%; subsequently, the results were used to calculate the standard deviation for each set of experimental conditions.

## 3. Results and Discussion

### 3.1. Solvent Effect on the HP-β-CD Particles

Our previous study showed that the vapor–liquid equilibrium (VLE) phase diagram of a CO_2_-water-ethanol ternary mixture can be used to qualitatively estimate the phase behavior of the mixtures in the saturator [17]. For instance, the feeding lines used a mass flow ratio of CO_2_ to 70.3% (*w/w*) aqueous ethanol solution (*m_CO_*_2_/*m_L_*) of 1.95, which was located within the two-phase region (H_2_O-rich liquid phase and CO_2_-rich vapor phase). The CO_2_ solubility in the aqueous liquid phase of the CO_2_-water-ethanol ternary system (with a mole fraction of approximately 0.18) undergoes a ninefold increase compared to that in the CO_2_-water binary system (with a mole fraction of <0.02) [20]. Therefore, enhanced atomization can be expected from aqueous ethanol solution containing dissolved CO_2_ with relatively low surface tension and low viscosity [21]. Finely atomized droplets resulted from the spray nozzle, followed by the production of fine solid particles after spray drying [22]. A similar strategy was applied in a new technique of supercritical assisted electrospraying (SA-ESPR) [23].

**Figure 1:**
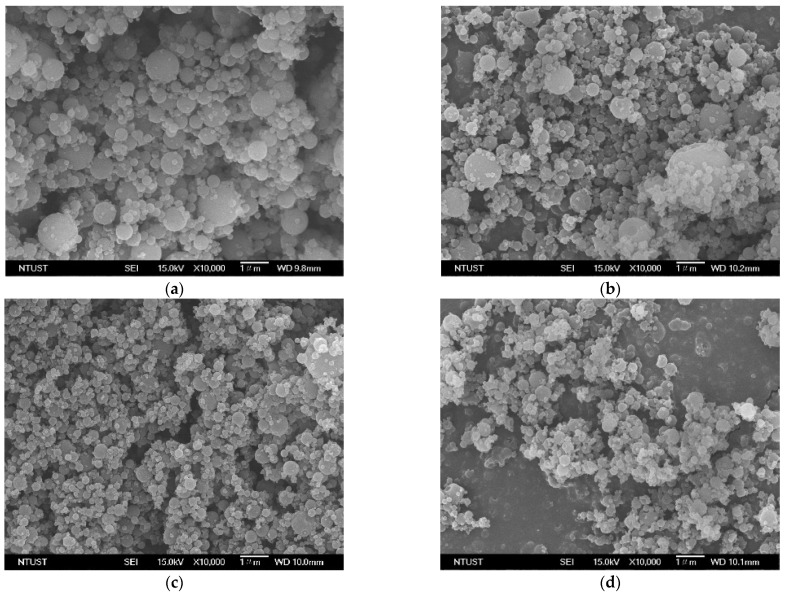

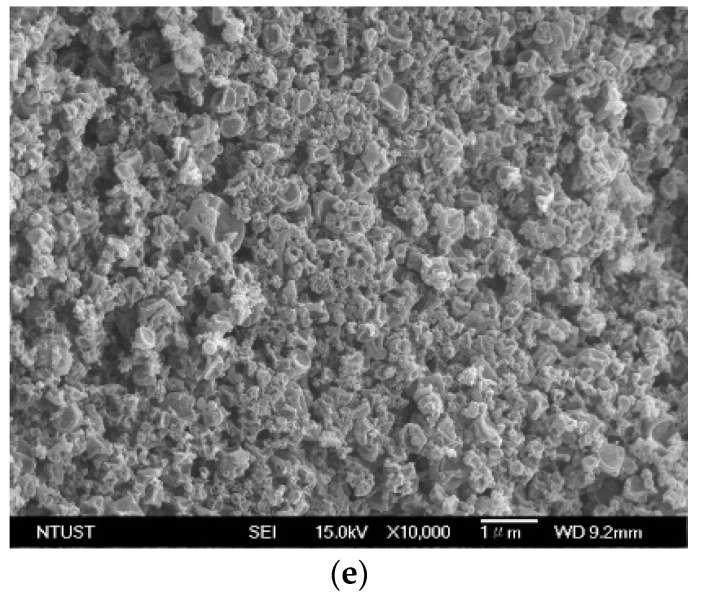
FESEM images of the HP-β-CD particles produced using the SAA process at different ethanol content of the HP-β-CD solution (EtOH%, *w/w*): (**a**) 0%; (**b**) 44.1%; (**c**) 54.2%; (**d**) 70.3%; and (**e**) 100%.

**Figure 2:**
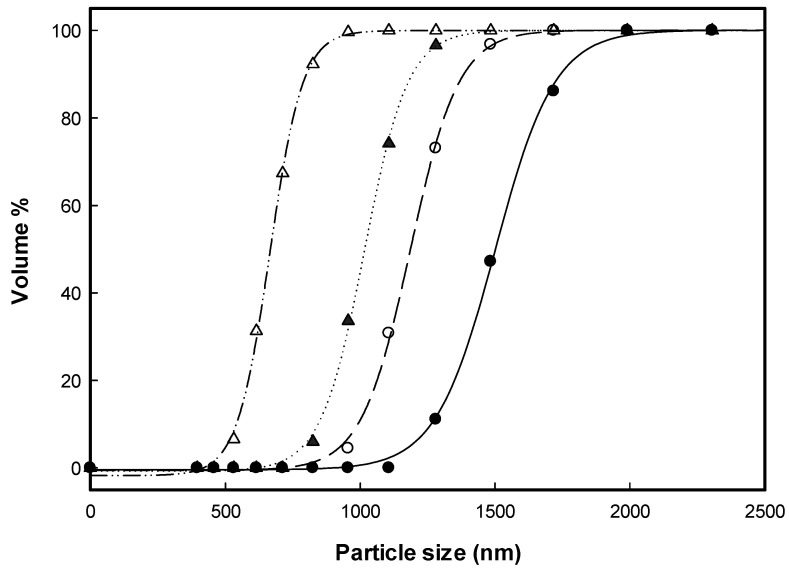
The *PSDs* of the HP-β-CD particles produced using the SAA at different ethanol content of the HP-β-CD solution (EtOH%, *w/w*): (•) 0%; (○) 44.1%; (▲) 54.2%; (△) 70.3%.

Table 1 lists the experimental conditions and results of the HP-β-CD particles produced using SAA. The experimental conditions included the ethanol content of the HP-β-CD solution (EtOH%, *w/w*), the temperatures of the precipitator (*T_P_*) and saturator (*T_S_*), the HP-β-CD concentration of the HP-β-CD solution (*C_HP_*), and the flow ratio of CO_2_ to HP-β-CD solution (*R*). Figure 1 and Figure 2 show the FESEM images and the *PSDs* of the HP-β-CD particles produced using the SAA process at different ethanol contents (0–100 EtOH%, *w/w*) of the HP-β-CD solution, respectively. As mentioned above, increasing the ethanol content of the HP-β-CD solution in SAA decreased the size of the HP-β-CD particles, which can be attributed to the enhanced atomization caused by the aqueous ethanol solution containing dissolved CO_2_ with low surface tension and low viscosity. Similar results have been reported for several materials [15,24,25]. The size of the HP-β-CD particles produced using pure ethanol as a solvent was not determined because of the uneven dispersion of the sample from the serious aggregation of irregular HP-β-CD particles (Figure 1e).

Figure 1 shows the solvent effect on the morphology of the HP-β-CD particles. Spherical HP-β-CD particles were produced using water as the solvent (Figure 1a) in the HP-β-CD solution, and irregularly shaped particles were produced with increasing ethanol content of the HP-β-CD solution. The Peclet number (*Pe*, the ratio of the evaporation rate to the diffusion coefficient of the solute) can be used to explain the particle formation process in spray drying. A small *Pe* indicates relatively slow evaporation of the solvent (e.g., water), which offers time for the solute to distribute homogeneously in the droplets, thereby producing spherical solid particles. In contrast, a high *Pe* indicates rapid evaporation of the solvent (e.g., ethanol) or slow diffusion of the solute from the surface, leading to shell or irregular particle formation [26]. Although increasing the ethanol content is beneficial for micronization, irregularly shaped particles can be easily produced, which is not favorable for the flowability of the resulting powder. To achieve both a spherical shape and micronization, a solution of 54.4% (*w/w*) aqueous ethanol was used as the solvent throughout the SAA to investigate the effect of the precipitation parameters on the HP-β-CD particle size.

### 3.2. Effects of the Precipitation Parameters on HP-β-CD Particle Size

**Figure 3:**
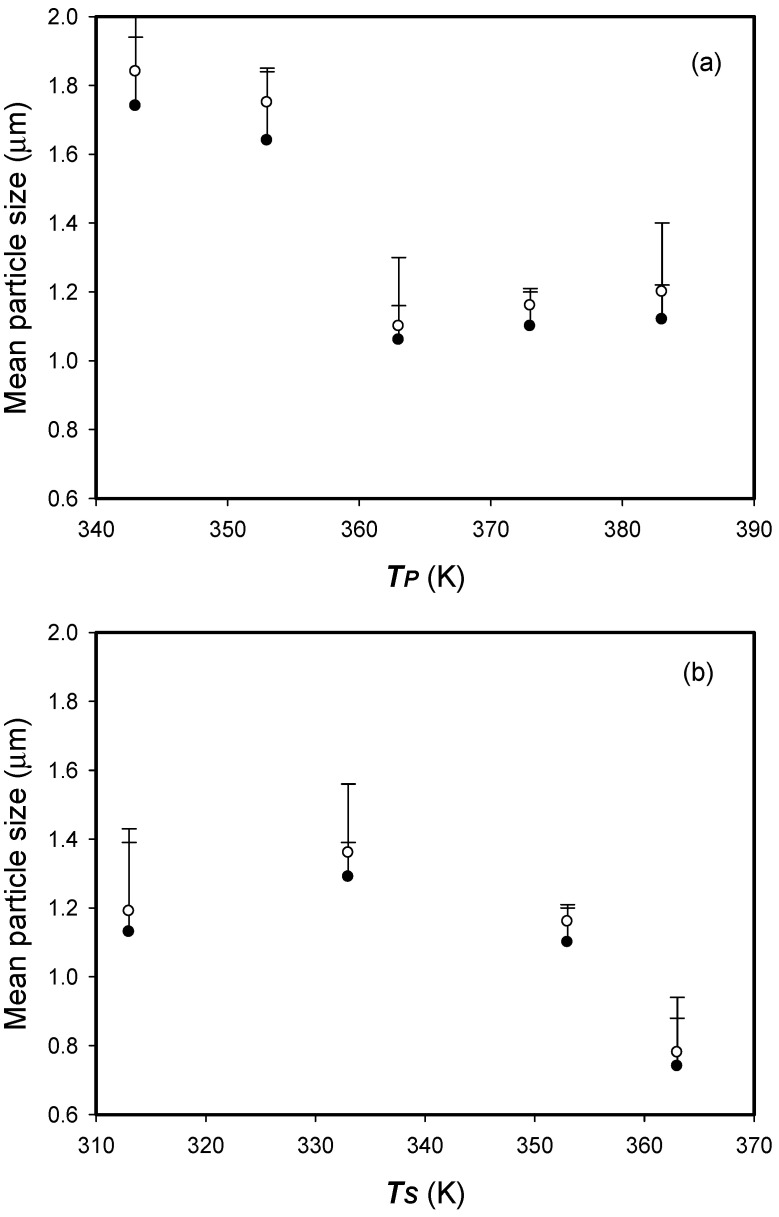
Arithmetic mean size (*d_no_*, •) and mass-weighted mean particle size (*d*_4,3_, ○) of the HP-β-CD particles varying with SAA process parameters of (**a**) the temperature of precipitator (*T_P_*) (**b**) the temperature of saturator (*T_S_*).

The effect of the temperatures of the precipitator (*T_P_*) and saturator (*T_S_*) on the mean particle size (*d*_4,3_) of the micronized HP-β-CD particle is shown in Figure 3 and was conducted using the following fixed conditions: a concentration (*C_HP_*) of 10 mg/mL and a volume flow ratio (*R*) of 1.8 (see Table 1, runs #2, #4−10). The decrease in the HP-β-CD particles size with increasing temperature is ascribed to the corresponding decrease in the viscosity of the solution liquid at the precipitator and saturator. A similar trend has been reported for other SAA processes [14,20,27]. Thus, the conducive conditions for producing fine and narrow distribution particles were precipitator temperature of 373.2 K and saturator temperature of 353.2 K.

**Figure 4:**
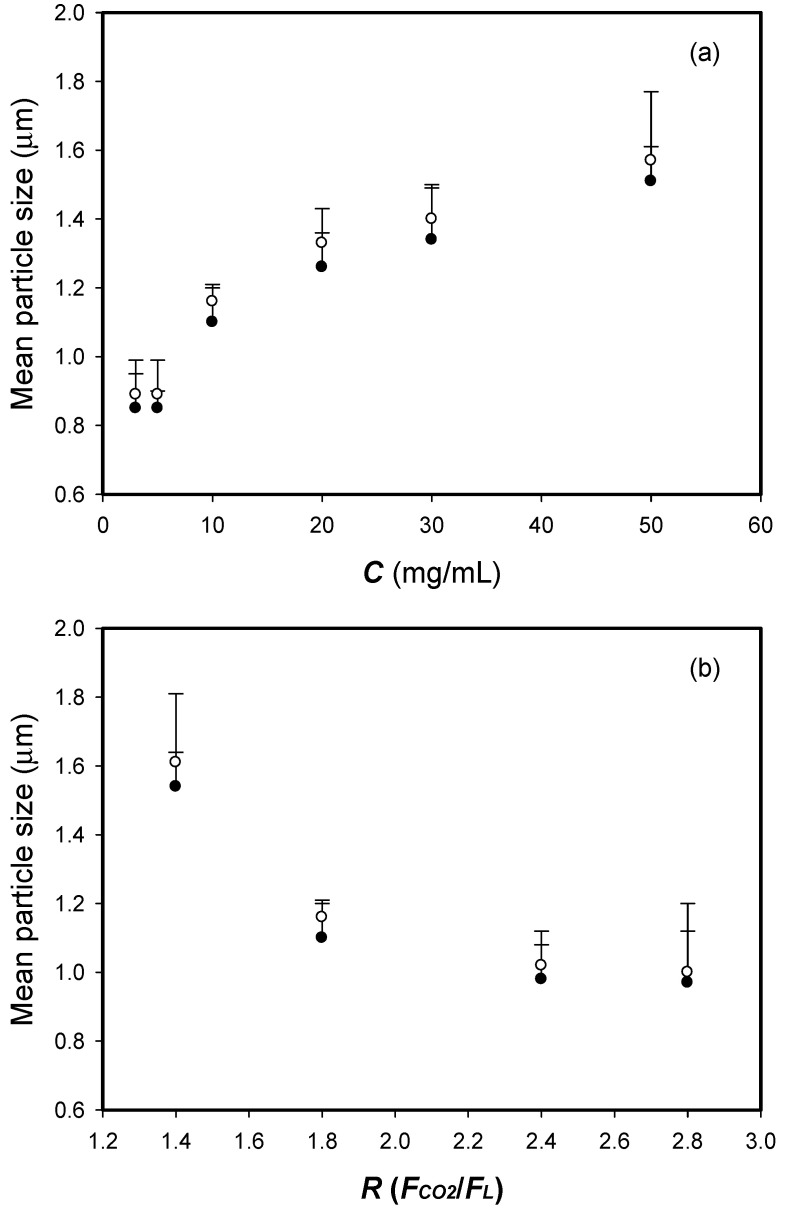
Arithmetic mean size (*d_no_*, •) and mass-weighted mean particle size (*d*_4,3_, ○) of the HP-β-CD particles varying with SAA process parameters of (**a**) the concentration of the HP-β-CD solution (*C_HP_*) (**b**) the volume flow ratio of CO_2_ to HP-β-CD solution liquid (*R*).

The effect of the concentration (*C_HP_*) of the HP-β-CD solution on the HP-β-CD particle size was examined in the range of 3 to 50 mg/mL (runs #2, and #11−15 in Table 1). Figure 4a indicates that the mean sizes of the HP-β-CD particles increased with the concentration of the HP-β-CD solution, which illustrates that the high viscosity of the HP-β-CD solution at high concentrations may result in large liquid droplets and increase the mean HP-β-CD particle size (*d*_4,3_) in SAA. Similar results have been reported, mostly for relevant SAA processes [14,15,20,24,25,27,28,29,30,31,32,33].

The mean particle size (*d*_4,3_) of the micronized HP-β-CD particles produced at different flow ratios (*R*) in the range of 1.4−2.8 (runs #2, and #16−18 in Table 1) are shown in Figure 4b. The mean HP-β-CD particle size (*d*_4,3_) decreased as the volume flow ratio (*R*) increased. A moderate excess of gas provides the energy necessary for liquid breakup and fine atomization during the SAA of an aqueous solution [28,34]. A similar trend was observed in polymer micronization via SAA [14,20,29] and for sodium cellulose sulfate micronization via an SAA-hydrodynamic cavitation mixer (HCM) process [27].

### 3.3. In Vitro Aerosolization Performance of HP-β-CD Carrier Particles with the Addition of _L_-Leucine

Several studies have reported that LEU has been added as a dispersion enhancer in the inhalable drug formulation [6,17,35,36]. We fixed the concentration of the HP-β-CD solution (*C_HP_*) at 10 mg/mL and used the abovementioned optimal SAA parameters (a precipitator temperature (*T_P_*) of 373.2 K, saturator temperature (*T_S_*) of 353.2 K, and flow ratio (*R*) of CO_2_ to HP-β-CD solution volume of 1.8) to investigate the aerosol performance of the HP-β-CD carrier particles with the addition of LEU. Table 2 lists the concentration of leucine in the HP-β-CD solution (*C_LEU_*) and the experimental results of in vitro aerosolization of the HP-β-CD carrier particles produced through SAA, including the mean particle sizes (*d*_4,3_) of the HP-β-CD carrier particles (HP-β-CD-LEU), in vitro aerodynamic properties in the form of *FPF* (%) and *MMAD* (μm), and flow properties in the form of the tapped density (*ρ_tap_*) and Hausner ratio (*H_R_* = *ρ_tap_*/*ρ_b_*).

**Figure 5:**
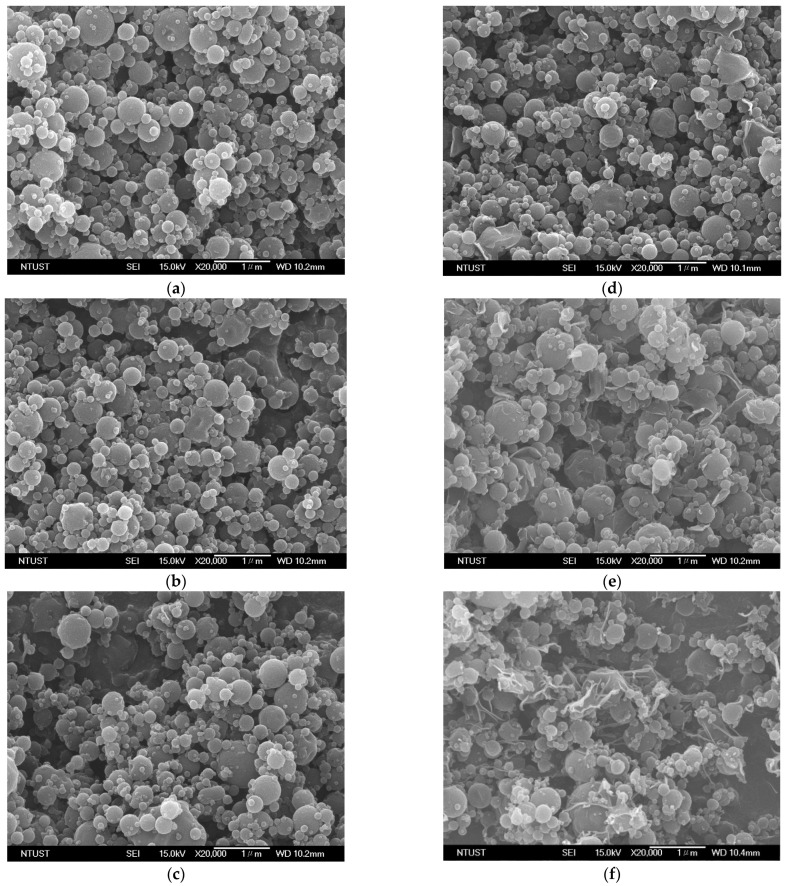
FESEM images of HP-β-CD carrier particles with differing addition of leucine (mass%) produced through SAA: (**a**) 0%, (**b**) 1%, (**c**) 4.8%, (**d**) 9.1%, (**e**) 13%, and (**f**) 16.7%.

Figure 5 shows the FESEM images of the HP-β-CD carrier particles with differing amounts of LEU (0, 1.0, 4.8, 9.1, 13.0, and 16.7 mass%, 100 × LEU/(LEU + HP-β-CD)) produced through SAA. The mean size of the HP-β-CD carrier particles was not significantly affected by the addition of LEU. However, the sample with 9.1 mass% LEU showed few tiny crystals on the surface of the HP-β-CD carrier particles (Figure 5d). Increased LEU content produces needle-like fibrous crystals on the surface of the HP-β-CD carrier particles. The wrinkled and rough surfaces become more pronounced with an increase in the LEU content. The same phenomenon was observed in our previous study of mannitol carrier particles [17] and Mohtar’s study of sulfobutylether-β-cyclodextrin complex particles [35].

**Figure 6:**
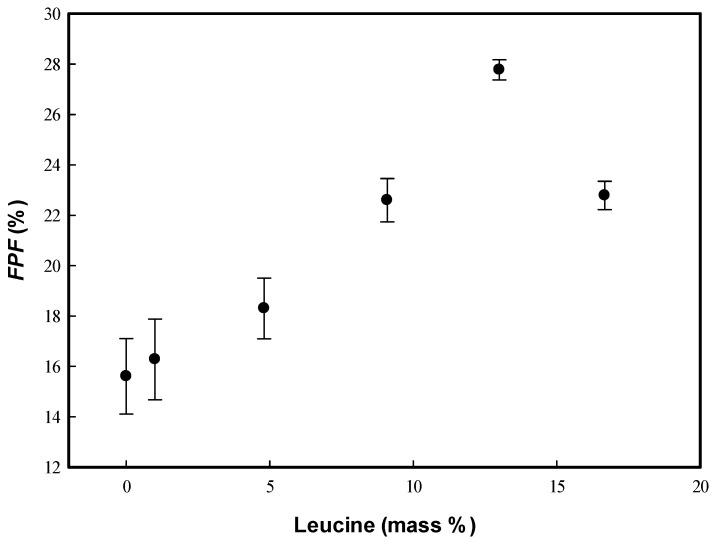
Fine particle fractions (*FPFs*) of the HP-β-CD carrier powder varying with the addition of leucine (mass%).

The in vitro aerodynamic results indicated that the *ED* fraction (*ED*, %) of the HP-β-CD carrier particle powder was higher than 95%, except in run #L6, and the *FPF* values increased with increasing LEU content in the HP-β-CD carrier samples (Figure 6). The *MMAD* of the HP-β-CD carrier particles decreases with increasing LEU, which indicated that LEU could function as a dispersion enhancer. Vartiainen’s study [6] showed similar results of dry powder inhalation formulations (corticosteroids, HP-β-CD, and leucine); a wrinkled morphology results from nanosized leucine crystals on the surface of drying particles and enhanced aerosolization performance concomitant with the lower contact area between particles. Several pulmonary drug delivery formulations exhibited enhanced aerosol behavior by decreasing the contact area and cohesion between the wrinkled particles [9,17,35,36,37,38].

Moreover, the in vitro aerosol performance could also be evaluated by a lower *H_R_*, which is indicative of the excellent flow properties of particulate powder [39,40]. The HP-β-CD particles with LEU exhibited better flowability (runs #L2−L5) than those without LEU (run #L1 exhibited high *H_R_* values or poor flow properties). HP-β-CD-LEU carrier particles exhibited excellent aerosol performance with the addition of 13 mass% LEU (optimal LEU addition, run #L5), which is comparable with the results in previous studies [17,41]. The *FPF* of the HP-β-CD carrier particles with the addition of 13 mass% LEU (run #L5) increased to 27.8 ± 0.4% (approximately double that of the HP-β-CD particles without LEU, run #L1), and the *MMAD* was as low as 2.32 ± 0.3 μm. However, when the LEU content increased to 16.7 mass% (run #L6), the FESEM image showed the presence of agglomerates (Figure 5f), which might account for the aerosol performance. If an excessive amount of LEU is added, the particle morphology might not favor the powder flowability and aerodynamic behavior (Figure 6). In summary, the aerodynamic performance of HP-β-CD carrier particles can be enhanced by the optimal addition of LEU.

### 3.4. Solid Characterization

**Figure 7:**
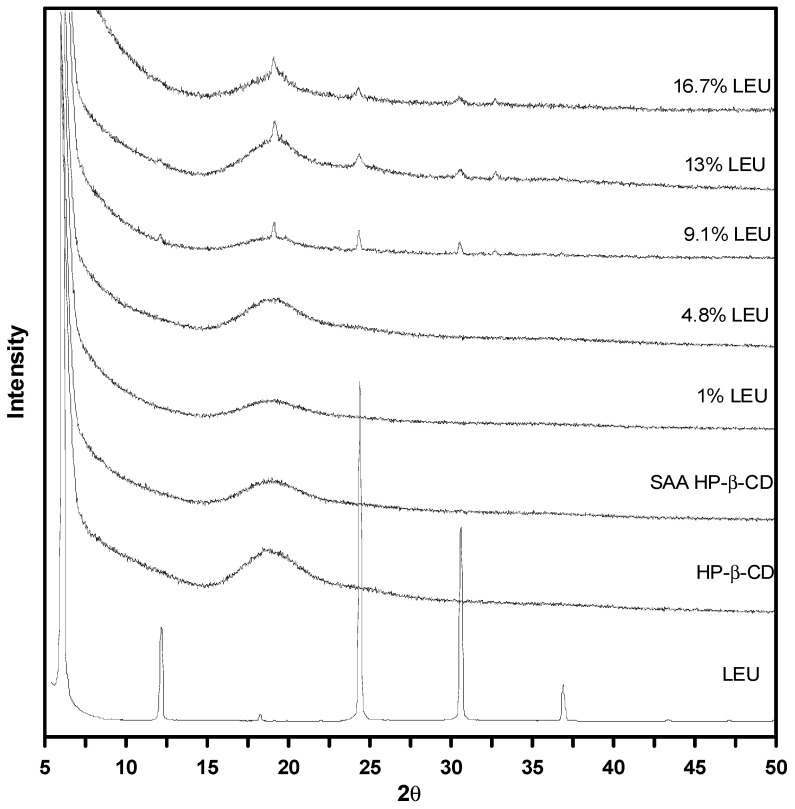
XRD patterns of the HP-β-CD carrier particles with varying amount of LEU (mass%) produced through SAA.

Figure 7 shows the XRD patterns for the HP-β-CD carrier particles with varying amount of LEU (mass%). A broad peak with a low intensity was recorded for the as-received HP-β-CD and HP-β-CD particles produced by SAA (denoted as SAA HP-β-CD in Figure 7), indicating their amorphous state. Interestingly, the samples of the HP-β-CD particles with LEU ≥9.1 mass% exhibited some characteristic diffraction peaks (20°, 24.4° and 30.6°) of the crystalline leucine, corresponding to the samples of runs #L4–L6. Further, we also observed the presence of crystalline LEU in the FESEM images of the HP-β-CD carrier particles (Figure 5d–f). The LEU crystal preferred the orientation of the peak at 20°, which was reported in the spray-drying process and is suggested to be a result of LEU crystallization at the surface of the HP-β-CD carrier particles [36,42,43].

**Figure 8:**
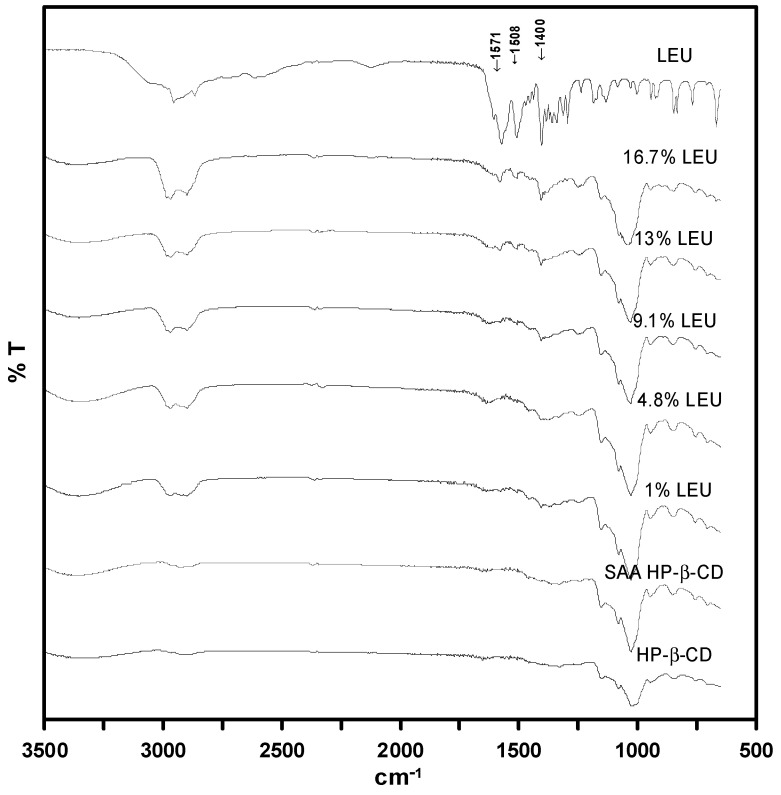
FTIR analysis spectra of the HP-β-CD carrier particles with varying amount of LEU (mass%) produced through SAA.

The FTIR analysis spectra (Figure 8) show the same patterns as those of the as-received HP-β-CD and HP-β-CD carrier particles produced by SAA. The spectrum of LEU shows an intense absorption peak at around 1571 cm^−1^, attributed to the COO– asymmetric stretching mode of vibration; the peak at 1508 cm^−1^ is indicative of the symmetric deformation of NH_3_^+^; and the symmetric stretching of the COO– ion group is observed at 1400 cm^−1^. The degree of recognition of these LEU peaks increased as the amount of LEU increased.

**Figure 9:**
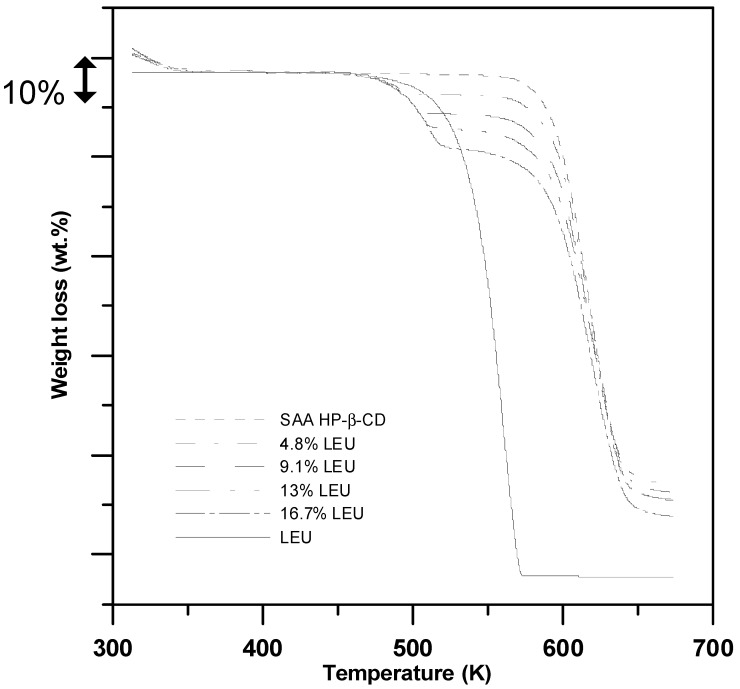
TGA analyses of the HP-β-CD carrier particles with varying amount of LEU (mass%) produced through SAA.

Figure 9 shows the thermogravimetric behavior of the HP-β-CD carrier particles with the addition of LEU (mass%) produced through SAA. The thermal behavior of the as-received leucine shows similar TGA measurements performed by Li et al. [44], wherein the leucine is heated, it sublimes and decomposes in the gas phase, and presents a one-stage weight loss (△m = 100%) between 473 and 573 K. The leucine content in the HP-β-CD carrier particles produced through SAA was determined by the weight loss difference (at 573 K in Figure 9) between the HP-β-CD carrier particles without leucine (SAA HP-β-CD) and HP-β-CD carrier particles with varying amount of LEU (4.8−16.7 mass%). The thermographs were artificially shifted to the same baseline to improve the readability of the graph. The TGA results showed that leucine contents in the HP-β-CD carrier particles with varying amount of LEU (4.8, 9.1, 13.0, and 16.7 mass%) were 5.1 ± 0.5%, 9.2 ± 0.3%, 13.1 ± 0.2%, and 16.9 ± 0.4%, respectively, and were consistent with the leucine formulations (*C_LEU_*) in Table 2.

## 4. Conclusions

Fine spherical HP-β-CD particles were successfully produced through SAA using aqueous ethanol as a solvent, and several crucial factors affecting the morphology and size of HP-β-CD particle were investigated. The optimal conditions for producing the fine spherical particles were determined to be 54.2% (*w/w*) aqueous ethanol as the solvent, high temperatures of the precipitator and saturator, high flow rate ratio of carbon dioxide to the HP-β-CD solution, and low concentrations of the HP-β-CD solution. LEU as a dispersion enhancer improved the aerosolization behavior of the HP-β-CD carrier particles by reducing the interparticle cohesion between the fine wrinkled surface particles and enhancing the aerodynamic performance of the HP-β-CD carrier particles with the optimal addition of 13 mass% LEU. This study suggested that the HP-β-CD carrier particles produced through SAA can be used in dry powder inhalation formulations. The investigation of the aerosol performance of DPI formulations of HP-β-CD and pulmonary delivery drugs is in progress.

## Figures and Tables

**Table 1:** Experimental conditions and results of the HP-β-CD particles produced using SAA.

Run	EtOH%	*T_P_*	*T_S_*	*C_HP_*	*R*	*d_no_*	*d*_4,3_
	*w/w*	K	K	mg/mL	*F_CO_*_2_/*F_L_*	μm	μm
1	44.1	373	353	10	1.8	1.28 ± 0.20	1.35 ± 010
2	54.2	373	353	10	1.8	1.10 ± 0.10	1.16 ± 0.05
3	70.3	373	353	10	1.8	0.72 ± 0.15	0.78 ± 0.20
4	54.2	343	353	10	1.8	1.74 ± 0.20	1.84 ± 0.20
5	54.2	353	353	10	1.8	1.64 ± 0.20	1.75 ± 0.10
6	54.2	363	353	10	1.8	1.06 ± 0.10	1.10 ± 0.20
7	54.2	383	353	10	1.8	1.12 ± 0.10	1.20 ± 0.20
8	54.2	373	313	10	1.8	1.13 ± 0.30	1.19 ± 0.20
9	54.2	373	333	10	1.8	1.29 ± 0.10	1.36 ± 0.20
10	54.2	373	363	10	1.8	0.74 ± 0.20	0.78 ± 0.10
11	54.2	373	353	3	1.8	0.85 ± 0.10	0.89 ± 0.10
12	54.2	373	353	5	1.8	0.85 ± 0.05	0.89 ± 0.10
13	54.2	373	353	20	1.8	1.26 ± 0.10	1.33 ± 0.10
14	54.2	373	353	30	1.8	1.34 ± 0.15	1.40 ± 0.10
15	54.2	373	353	50	1.8	1.51 ± 0.10	1.57 ± 0.20
16	54.2	373	353	10	1.4	1.54 ± 0.10	1.61 ± 0.20
17	54.2	373	353	10	2.4	0.98 ± 0.10	1.02 ± 0.10
18	54.2	373	353	10	2.8	0.97 ± 0.15	1.00 ± 0.20

**Table 2:** In vitro aerosolization experiment results of HP-β-CD carrier particles with the addition of leucine.

Run	*C_LEU_*	*ED*	*FPF*	*MMAD*	*d_no_*	*d*_4,3_	*ρ_tap_*	*H_R_*
	mg/mL	%	%	µm	µm	µm	g/cm^3^	*ρ_tap_*/*ρ_b_*
L1	0	95.4 ± 1.2	15.6 ± 1.5	9.42 ± 0.60	1.10 ± 0.05	1.16 ± 0.09	0.24 ± 0.03	1.40 ± 0.05
L2	0.1	97.0 ± 1.2	16.3 ± 1.6	7.06 ± 0.80	1.11 ± 0.10	1.20 ± 0.08	0.22 ± 0.03	1.29 ± 0.04
L3	0.5	96.8 ± 0.1	18.3 ± 1.2	8.39 ± 0.50	1.27 ± 0.12	1.32 ± 0.10	0.23 ± 0.03	1.35 ± 0.04
L4	1.0	96.2 ± 0.1	22.6 ± 0.9	5.35 ± 0.80	1.32 ± 0.12	1.45 ± 0.06	0.22 ± 0.04	1.32 ± 0.03
L5	1.5	96.6 ± 0.0	27.8 ± 0.4	2.32 ± 0.30	1.34 ± 0.06	1.42 ± 0.05	0.23 ± 0.03	1.33 ± 0.03
L6	2.0	82.9 ± 0.2	22.8 ± 0.6	2.52 ± 0.40	1.36 ± 0.14	1.43 ± 0.13	0.23 ± 0.04	1.40 ± 0.10

## Data Availability

Not applicable.

## References

[1] Brewster M.E., Loftsson T. (2007). Cyclodextrins as pharmaceutical solubilizers. Adv. Drug Deliv. Rev..

[2] Challa R., Ahuja A., Ali J., Khar R.K. (2005). Cyclodextrins in drug delivery: An updated review. AAPS PharmSciTech.

[3] Chelly J.E., Lacouture P.G., Reyes C.R.D. (2018). Safety of injectable HPbCD-diclofenac in older patients with acute moderate-to-severe postoperative pain: A pooled analysis of three phase III trials. Drugs Aging.

[4] Matilainen L., Toropainen T., Vihola H., Hirvonen J., Järvinen T., Jarho P., Järvinen K. (2008). In vitro toxicity and permeation of cyclodextrins in Calu-3 cells. J. Control. Release.

[5] Amaro M.I., Tajber L., Corrigan O.I., Healy A.M. (2015). Co-spray dried carbohydrate microparticles: Crystallisation delay/inhibition and improved aerosolization characteristics through the incorporation of hydroxypropyl-β-cyclodextrin with amorphous raffinose or trehalose. Pharm. Res..

[6] Vartiainen V., Bimbo L.M., Hirvonen J., Kauppinen E.I., Raula J. (2017). Aerosolization, drug permeation and cellular interaction of dry powder pulmonary formulations of corticosteroids with hydroxypropyl-β-cyclodextrin as a solubilizer. Pharm. Res..

[7] Dufour G., Bigazzi W., Wong N., Boschini F., Tullio P., Piel G., Cataldo D., Evrard B. (2015). Interest of cyclodextrins in spray-dried microparticles formulation for sustained pulmonary delivery of budesonide. Int. J. Pharm..

[8] Healy A.M., Amaro M.I., Paluch K.J., Tajber L. (2014). Dry powders for oral inhalation free of lactose carrier particles. Adv. Drug Deliv. Rev..

[9] Suzuki É.Y., Amaro M.I., de Almeida G.S., Cabral L.M., Healy A.M., de Sousa V.P. (2018). Development of a new formulation of roflumilast for pulmonary drug delivery to treat inflammatory lung conditions. Int. J. Pharm..

[10] Tozuka Y., Wongmekiat A., Sakata K., Moribe K., Oguchi T., Yamamoto K. (2004). Co-grinding with cyclodextrin as a nanoparticle preparation method of a poorly water soluble drug. J. Incl. Phenom. Macrocycl. Chem..

[11] Kreaz A.R.M., Abu-Eida E.Y., Erő I., Kata M. (1999). Freeze-dried complexes of furosemide with cyclodextrin derivatives. J. Incl. Phenom. Macrocycl. Chem..

[12] Yurtdaş G., Demirel M., Genҫ L. (2011). Inclusion complexes of fluconazole with β-cyclodextrin: Physicochemical characterization and in vitro evaluation of its formulation. J. Incl. Phenom. Macrocycl. Chem..

[13] Reverchon E., Adami R., Caputo G. (2006). Supercritical assisted atomization: Performance comparison between laboratory and pilot scale. J. Supercrit. Fluids.

[14] Wu H.T., Yang M.W. (2011). Precipitation kinetics of PMMA sub-micrometric particles with a supercritical assisted-atomization process. J. Supercrit. Fluids.

[15] Wu H.T., Huang S.C., Yang C.P., Chien L.J. (2015). Precipitation parameters and the cytotoxicity of chitosan hydrochloride microparticles production by supercritical assisted atomization. J. Supercrit. Fluids.

[16] Reverchon E., Antonacci A. (2006). Cyclodextrins micrometric powders obtained by supercritical fluid processing. Biotechnol. Bioeng..

[17] Wu H.-T., Li T.-H., Tsai H.-M., Chien L.-J., Chuang Y.-H. (2021). Formulation of inhalable beclomethasone dipropionate-mannitol composite particles through low-temperature supercritical assisted atomization. J. Supercrit. Fluids.

[18] Day C.P.F., Miloserdov A., Wildish-Jones K., Pearson E., Carruthers A.E. (2020). Quantifying the hygroscopic properties of cyclodextrin containing aerosol for drug delivery to the lungs. Phys. Chem. Chem. Phys..

[19] O’Shaughnessy P.T., Raabe O.G. (2003). A comparison of cascade impactor data reduction methods. Aerosol Sci. Technol..

[20] Wu H.T., Yang M.W., Huang S.C. (2014). Sub-micrometric polymer particles formation by a supercritical assisted-atomization process. J. Taiwan Inst. Chem. Eng..

[21] Wang X.F., Lefebvre A.H. (1987). Mean drop sizes from pressure-swirl nozzles. J. Propul. Power.

[22] Kawakami K., Sumitani C., Yoshihashi Y., Yonemochi E., Terada K. (2010). Investigation of the dynamic process during spray-drying to improve aerodynamic performance of inhalation particles. Int. J. Pharm..

[23] Baldino L., Cardea S., Reverchon E. (2019). Supercritical assisted electrospray: An improved micronization process. Polymers.

[24] Reverchon E. (2002). Supercritical-assisted atomization to produce micro- and/or nanoparticles of controlled size and distribution. Ind. Eng. Chem. Res..

[25] Wu H.T., Su Y.C., Wang Y.M., Tsai H.M. (2018). Characterization and aerosolization performance of mannitol particles produced using supercritical assisted atomization. Chem. Eng. Res. Des..

[26] Vehring R. (2008). Pharmaceutical particle engineering via spray drying. Pharm. Res..

[27] Wang Q., Guan Y.X., Yao S.J., Zhu Z.Q. (2010). Microparticle formation of sodium cellulose sulfate using supercritical fluid assisted atomization introduced by hydrodynamic cavitation mixer. Chem. Eng. J..

[28] Reverchon E., Antonacci A. (2006). Chitosan microparticles production by supercritical fluid processing. Ind. Eng. Chem. Res..

[29] Reverchon E., Antonacci A. (2007). Polymer microparticles production by supercritical assisted atomization. J. Supercrit. Fluids.

[30] Reverchon E., Spada A. (2004). Erythromycin micro-particles produced by supercritical fluid atomization. Powder Technol..

[31] Cai M.-Q., Guan Y.-X., Yao S.-J., Zhu Z.-Q. (2008). Supercritical fluid assisted atomization introduced by hydrodynamic cavitation mixer (SAA-HCM) for micronization of levofloxacin hydrochloride. J. Supercrit. Fluids.

[32] Wu H.T., Yang M.W. (2012). Precipitation kinetics of PMMA-co-BMA sub-micrometric particles with compressed CO_2_ assisted-atomization process. Powder Technol..

[33] Wu H.T., Lee H.K., Chen H.C., Chien L.J. (2015). Precipitation kinetics and biological properties of chitosan microparticles produced using supercritical assisted atomization. Chem. Eng. Res. Des..

[34] Caputo G., Liparoti S., Adami R., Reverchon E. (2012). Use of supercritical CO_2_ and N_2_ as dissolved gases for the atomization of ethanol and water. Ind. Eng. Chem. Res..

[35] Mohtar N., Taylor K.M.G., Sheikh K., Somavarapu S. (2017). Design and development of dry powder sulfobutylether-β-cyclodextrin complex for pulmonary delivery of fisetin. Eur. J. Pharm. Biopharm..

[36] Lamy B., Serrano D.R., O’Connell P., Couet W., Marchand S., Healy A.M., Tewes F. (2019). Use of leucine to improve aerodynamic properties of ciprofloxacin loaded maltose microparticles for inhalation. Eur. J. Pharm. Res..

[37] Seville P.C., Learoyd T.P., Li H.Y., Williamson I.J., Birchall J.C. (2007). Amino acid-modified spray-dried powders with enhanced aerosolisation properties for pulmonary drug delivery. Powder Technol..

[38] Sou T., Kaminskas L.M., Nguyen T.H., Carlberg R., Mclntosh M.P. (2013). The effect of amino acid excipients on morphology and solid-state properties of multi-component spray-dried formulations for pulmonary delivery of biomacromolecules. Eur. J. Pharm. Biopharm..

[39] Abdullah E.C., Geldart D. (1999). The use of bulk density measurements as flowability indicators. Powder Technol..

[40] Saw H.Y., Davies C.E., Paterson A.H.J., Jones J.R. (2015). Correlation between powder flow properties measured by shear testing and Hausner ratio. Procedia Eng..

[41] Molina C., Kaialy W., Nokhodchi A. (2019). The crucial role of leucine concentration on spray dried mannitol-leucine as a single carrier to enhance the aerosolization performance of albuterol sulfate. J. Drug Deliv. Sci. Technol..

[42] Raula J., Kuivanen A., Lähde A., Jiang H., Antopolsky M., Kansikase J., Kauppinen E.I. (2007). Synthesis of L-leucine nanoparticles via physical vapor deposition at varying saturation conditions. J. Aerosol Sci..

[43] Li L., Leung S.S.Y., Gengenbach T., Yu J., Gao G., Tang P., Zhou Q., Chan H.-K. (2017). Investigation of _L_-leucine in reducing the moisture-induced deterioration of spray-dried salbutamol sulfate power for inhalation. Int. J. Pharm..

[44] Li J., Wang Z., Yang X., Hu L., Liu Y., Wang C. (2006). Decomposing or subliming? An investigation of thermal behavior of l-leucine. Thermochim. Acta.

